# The Hypolipidemic Activity of Metal Complexes of Amine Carboxyboranes in Rodents

**DOI:** 10.1155/MBD.1994.329

**Published:** 1994

**Authors:** Iris H. Hall, Bernard F. Spielvogel, Anup Sood, Karan W. Morse, Verrill M. Norwood III, Oi. T. Wong

**Affiliations:** 1 Division of Medicinal Chemistry and Natural Products School of Pharmacy C.B. 7360 University of North Carolina Chapel Hill N.C. 27559-7360 USA; 2 Boron Biologicals, Inc. 533 Pylon Dr. Raleigh N.C. 27606 USA; 3 Department of Chemistry and Biochemistry Utah State University Logan Utah 84322 USA

## Abstract

The metal complexes of amine-carboxyborane including copper, chromium, zinc, calcium amd cobalt were effective hypolipidemic agents lowering both serum cholesterol and triglyceride levels significantly in mice at 8 mg/kg/day, I.P. after 16 days. The agents reduced acetyl CoA synthetase, ATP-dependent citrate lyase, acyl CoA cholesterol acyl transferase, sn-glycerol-3-phosphate acyl transferase activities of rat liver and small intestinal mucosa after 14 days treatment. The neutral cholesterol ester hydrolase activity was elevated by the agents in both tissues. The metal complexes altered lipid levels in the bile of rats after treatment as well as the bile acid composition after 14 days administration, orally. The agents blocked enterohepatic absorption of cholesterol from rat isolated intestinal loops.


### THE HYPOLIPIDEMIC ACTIVITY OF METAL COMPLEXES OF AMINE CARBOXYBORANES IN RODENTS

Iris H. Hall[1], Bernard F. Spielvogel[2], Anup Sood[2], Karan W. Morse[3], Verrill M. Norwood III[3] and Oi. T. Wong[1]

[1]. Division of Medicinal Chemistry and Natural Products, School of Pharmacy, C.B. 7360, University of North Carolina, Chapel Hill, N.C. 27559-7360, USA

[2]. Boron Biologicals, Inc., 533 Pylon Dr. Raleigh, N.C. 27606, USA

[3]. Department of Chemistry and Biochemistry, Utah State University, Logan, Utah 84322, USA

### ABSTRACT

The metal complexes of amine-carboxyborane including copper, chromium, zinc, calcium amd cobalt were effective hypolipidemic agents lowering both serum cholesterol and triglyceride levels significantly in mice at 8 mg/kg/day, I.P. after 16 days. The agents reduced acetyl CoA synthetase, ATP-dependent citrate lyase, acyl CoA cholesterol acyl transferase, sn-glycerol-3-phosphate acyl transferase activities of rat liver and small intestinal mucosa after 14 days treatment. The neutral cholesterol ester hydrolase activity was elevated by the agents in both tissues. The metal complexes altered lipid levels in the bile of rats after treatment as well as the bile acid composition after 14 days administration, orally. The agents blocked enterohepatic absorption of cholesterol from rat isolated intestinal loops.

### INTRODUCTION

Amine-carboxyboranes have proven to be effective hypolipidemic agents in mice I.P. and rats, orally at 5-20 mg/kg/day [1-4]. Both serum cholesterol and triglyceride levels are significantly reduced. These agents successfully reduced VLDL and LDL cholesterol levels and elevated HDL cholesterol levels in rats. One of the modes of action of the derivatives was the suppression of rate limiting enzyme activities of de novo synthesis of lipids. One particular derivative tetrakis-u- (trimethylamine-boranecarboxylato)-bis(trimethylamine-carboxyborane)-dicopper (II), compound 1, demonstrated excellent activity at 2.5 mg/kg/day [5]. Preliminary studies suggested that this derivative increase lipid excretion in the bile of rats [5]. Thus, the present study extends the investigation of metal complexes of amine-carboxyboranes as hypolipidemic agents and their role on biliary lipid excretion in rodents.

### MATERIALS AND METHODS

Source of Material and Compounds

The synthesis and physical characteristics of these complexes has previously been reported: $Cu(u\text{-}(CH_3)_3NBH_2COO)_4.2(CH_3)_3NBH_2COOH$ **1** [6] $[Fe_3O((CH_3)_3NBH_2COO)_6(CH_3OH)]NO_3.CH_3CN$ **2** [7], $[Fe_3O((CH_3)_3NBH_2COO)_6(CH_3OH)_3]Cl$ **3** [7], $[Cr_3O((CH_3)_3NBH_2COO)_6(H_2O)_3]NO_3.CH_3OH.CH_3CN$ **4** [7], cis$[Co(en)_2((CH_3)_3NBH_2COO)_2]Cl\cdot 2.5\ H_2O\cdot 0.5\ CH_3OH$ **5**, [8], $ZnCl_2.2\ (CH_3)_3NBH_2CN$ **6** [9], $Ca((CH_3)_3\ N\cdot BH_2COO)\ NO_3\cdot CH_3\ COCH_3\cdot 0.5H_2O$ **7** [8], $Na(NH_3.BH_2CN)_6I$ **8** [10], $Na(CH_3)_3NBH_2COO\cdot 0.25CH_3OH$ **9** [7], $Na(CH_3)_2\ NBH_2\ COO\cdot 0.45H_2O$ **10** [7], $Na(CH_3)_2NBH_3$ **11** [commercially available], $NaBH_3CN$ 12, $Na_2BH_3COO$ **13**, and $Na_2B_{10}H_{10}\cdot H_2O$ **14** [11]. Drugs were prepared in 1% carboxymethylcellulose and homogenized. Control animals were maintained on 1% carboxymethylcellulose as a vehicle treated group.

All isotopes were purchased from New England Nuclear. Substrates and cofactors were obtained from Sigma Chemical Co., and HPLC column and eluants were obtained from Waters Millipore Co.

Hypolipidemic Screen in Mice

Test compounds were administered to CF-1 male mice (28g) at 8 mg/kg/day I.P, for 16 days. On days 9 and 16, blood was obtained by tail-vein bleeding and the serum was obtained by centrifugation at 3500 g x 10 min. The serum cholesterol levels were determined by a modification of the Liebermann-Burchard reaction [12]. Serum triglyceride were determined using a commercial kit [Boehringer Mannheim Diagnostics]. Sprague Dawley male rats (~ 280g) were administered compound **1** at 2.5 mg/kg/day, orally and compound **4** at 8 mg/kg/day orally. Blood was collected on day 14 for serum lipid analysis. These doses were selected based on a preliminary pilot study for the best efficacy of the indivivdual agents in rats.

Treatment of Rats

Bile Cannulation Studies. Sprague Dawley male rats (~300g) were administered compounds **1** at 2.5 mg/kg/day or 4 at 8 mg/kg/day by intubation tube, orally for 14 days. The rats were administered chlorpromazine (25mg/kg) and anesthetized 30 minutes later with pentobarbital (22 mg/kg i.p.) [13], a combination which reduces the amount of pentobarbital necessary to maintain anesthesia level over extended periods of time without bronchial spasms. An incision was made just below the rib cage to expose the stomach and the duodenum. Once the bile duct was indentified, a loose ligature was placed around it and the duct was knicked. PE-10 plastic tubing was placed in the duct and tied in place. The bile was collected from the control and treated groups over the next 6 hours while the animals were maintained under anesthesia.

Bile Lipid Levels. The flow rate of the bile was calculated for each group as ml/min. Aliquots were extracted for lipids by the Folch et al. [14] and Bligh and Dyer [15] methods. Cholesterol [12], triglyceride [Bio-Dynamics/bmc triglyceride kit], neutral lipids [16], and phospholipid contents [17] were determined. Protein concentrations were also determined [18].

HPLC Analysis of Bile Acids Content. Samples were frozen overnight and thawed. The bile lipids were extracted with EtOH:water; [1:20] and filtered [19, 20]. Aliquots (250 μl) of the bile were added to 10 μl of the internal standard (testosterone). Then 4.75 ml of hot analytical grade ethanol was added, vortexed, and allowed to evaporate in a boiling water bath. The residues were dissolved in 250 μl of the mobile phase A [acetonitrile-methanol-0.03 M phosphate buffer, pH 3.4 (10:60:30 v/v/v)] and 100 μl was injected onto the HPLC column (μ Bondapak C18 column) (30 cm x 3.9 mm ID) (Waters) with a guard column (Bondapak C18/Corasi) (Waters) eluted with mobile phase A. The flow rate was 0.5 ml/min (600 psi isobaric flow) with detection at 210 nm. Standard bile acids (Sigma Chemical Co.) were purchased and were treated identical as the bile biological samples.

Absorption from In Situ Duodenum Loops. Sprague Dawley male rats were treated at 8 mg/kg/day orally and anesthesized as indicated above and the duodenum was isolated and knicked. Glass L-shaped cannulaes were placed at the proximal end of the duodenum and 20 cm distally down the intestine [21]. The segment was perfused with isotonic PBS buffer, pH 7.2 until the lumen was clear and all material expelled. Drug solution (0.2 ml) (20 mg/kg) was placed in the loop with either 1, 2-3H- cholesterol (54.8 Ci/mol) or 2,4-3H- cholic acid (25 mCi/mol) (2μCi). Aliquots (50 ml) were periodically removed over the next 210 minutes and the radioactivity determined using a Packard scintillation counter after correcting for quenching. The disappearance of the isotope from the loop over time was plotted for the control and treated animals.

Hepatic and Small Intestinal Enzymatic Studies. Sprague Dawley male rats (~280 g) were administered drugs 1 at 2.5 mg/kg/day, or 4 at 8 mg/kg/day, orally for 14 days. On day 15, the animals were sacrificed and the liver and small intestinal mucosa were excised. Homogenates (10%) in 0.25 M sucrose + 0.001 M EDTA, pH 7.2 were prepared of the liver and small intestinal mucosa [22]. The following enzyme assays were determined by literature techniques: ATP dependent citrate lyase [23], acetyl CoA synthetase [24], HMG-CoA reductase [25,26], acyl-CoA cholesterol acyl transferase [27], neutral cholesterol ester hydrolases [28], cholesterol-7a hydroxylase [29], acetyl CoA carboxylase [30], sn-glycerol-3-phosphate acyl transferase [31], phosphatidylate phosphohydrolase [32] and lipoprotein lipase [33].

Statistical Analysis

Data displayed in Tables 1-4 represent means ± standard deviations. The Student's "t" test was applied between control groups and the individual drug treatment groups. The analysis of variance (ANOVA) was applied among test drugs and is reported in the text only.

### RESULTS

The calcium (7) and the chromium (4) complexes demonstrated the best hypolipidemic acivity in mice on day 16 after dosing at 8 mg/kg/day causing greater than 50% reduction of serum cholesterol levels [Table 1]. The two iron complexes 2 and 3 and the sodium salts 9 and 14 caused at least 40% reduction of serum cholesterol levels after 16 days administration. The serum triglyceride levels on day 16 were reduced most significantly, i.e. greater than 50% by compounds **1** and **3**. Compounds **2, 4, 6, 12, 13** and **15** caused at least 40 % reduction of serum triglyceride levels in mice. In rats treated with compound **1** at 2.5 mg/kg/day, the serum cholesterol levels on days 14 were reduced 49% and serum triglyceride levels were reduced 60% compared to the control values. Compound **4** at 8 mg/kg/day reduced cholesterol values 51% and triglyceride levels 46% after 14 days.
In rats treated with compound **1** at 2.5 mg/kg/day liver enzyme activities for acetyl CoA synthetase, ATP-dependent citrate lyase, acyl CoA cholesterol acyl transferase, cholesterol-7-a hydroxylase, sn-glycerol-3-phosphate acyl transferase and phosphatidylate phosphohydrolase were all inhibited after 14 days dosing [Table 2]. In the small intestinal mucosa after treatment with compound 1 the activities of acetyl CoA synthetase, ATP- dependent citrate lyase, cholesterol-7-a hydrolase, acyl CoA cholesterol acyl transferase, neutral cholesterol ester hydrolase, phosphatidylate phosphohydrolase and lipoprotein lipase were inhibited significantly. Liver neutral cholesterol ester hydrolase and lipoprotein lipase activities were elevated after 14 days treatment.

Compound 4 after treatment in vivo at 8 mg/kg/day for 14 days significantly reduced liver acetyl CoA synthetase, ATP-dependent citrate lyase, HMG CoA reductase, cholesterol 7-a-

TABLE 1
The Hypolipidemic Activity of Metal Complexes of Amine-Carboxyboranes and Related Compounds in CF-1 Male Mice Administered at 8 mg/kg/day I.P.

| | Percent of Control [X ± S.D.] | | |
|---|---|---|---|
| | Serum Cholesterol | | Serum Triglyceride |
| Compounds | Day 9 | Day 16 | Day 16 |
| Control 1% CMS | 100 ± 6[a] | 100 ± 5[b] | 100 ± 7[c] |
| 1 | 71 ± 6* | 63 ± 5* | 47 ± 5* |
| 2 | 58 ± 5* | 59 ± 4* | 54 ± 6* |
| 3 | 74 ± 7 | 55 ± 5* | 48 ± 6* |
| 4 | 68 ± 6* | 45 ± 4* | 55 ± 4* |
| 5 | 89 ± 7 | 60 ± 5* | 65 ± 3* |
| 6 | 85 ± 5 | 71 ± 6 | 50 ± 5* |
| 7 | 80 ± 6 | 49 ± 4* | 67 ± 6* |
| 8 | 88 ± 7 | 64 ± 5* | 72 ± 5* |
| 9 | 56 ± 4* | 53 ± 3* | 70 ± 6* |
| 10 | 108 ± 6 | 63 ± 5* | 72 ± 5* |
| 11 | 103 ± 6 | 77 ± 7* | 89 ± 7 |
| 12 | 74 ± 6* | 74 ± 5* | 59 ± 5* |
| 13 | 76 ± 4* | 74 ± 6* | 57 ± 6* |
| 14 | 80 ± 5 | 56 ± 5* | 81 ± 5* |
| 15 | 61 ± 6 | 61 ± 5* | 53 ± 5* |

a = 125 mg% b = 128 mg% c = 137 mg% * = $P \leq 0.001$ Student's "t" test.

hydroxylase, acyl CoA cholesterol acyl transferase and sn-glycerol-3-phosphate acyl transferase activities [Table 3].

The compound reduced small intestinal mucosa ATP-dependent citate lyase, cholesterol-7-a hydroxylase, acyl CoA cholesterol acyl transferase, sn-glycerol-3-phosphate acyl transferase, and lipoprotein lipase activities. Liver and small intestine neutral cholesterol ester hydrolase, and liver lipoprotein lipase activities were increased after dosing with compound **4** after 14 days.

In rats treated for 14 days with compound **1** the bile flow rate was increased whereas with compound **4** the flow rate was decreased compared to the control value [Table 4]. Compound **1** increased biliary triglyceride levels to 237% of the control and all biliary

TABLE 2

The Effects of Compound 1 on Sprague Dawley Rat Liver and Small Intestinal Mucosa Enzyme Activities After 14 Day Oral Administration at 2.5 mg/kg/day orally.

| Percent of Control [X ± S.D.] | | | | |
|---|---|---|---|---|
| Enzyme Assayed | Liver | | Small Intestinal Mucosa | |
| N = 6 | Control | Treated | Control | Treated |
| Acetyl CoA synthetase | 100 ± 5[a] | 33 ± 5* | 100 ± 6[b] | 66 ± 5* |
| | 33 ± 5* | | | |
| | 100 ± 6[b] | | | |
| ATP-dep't Citrate Lyase | 100 ± 4[c] | 25 ± 6* | 100 ± 5[d] | 32 ± 7 |
| HMG CoA reductase | 100 ± 6[e] | 94 ± 6 | 100 ± 7[f] | 93 ± 6 |
| Acyl CoA Chol.Acyl Trans | 100 ± 5[g] | 34 ± 2* | 100 ± 4[h] | 45 ± 4* |
| Cholesterol-7-hydroxylase | 100 ± 3[i] | 54 ± 4* | 100 ± 4[j] | 18 ± 2* |
| Chol. Ester Hydrolase | 100 ± 4[k] | 135 ± 7* | 100 ± 5[l] | 63 ± 4* |
| Acetyl CoA carboxylase | 100 ± 4[m] | 84 ± 6 | 100 ± 6[n] | 83 ± 9 |
| sn-Glycerol-3-phosphate acyl transferase | 100 ± 5[o] | 34 ± 3* | 100 ± 4[p] | 88 ± 6 |
| Phosphatidylate phosphohydrolase | 100 ± 6[q] | 27 ± 6* | 100 ± 6[r] | 56 ± 5* |
| Lipoprotein Lipase | 100 ± 5[s] | 127 ± 4* | 100 ± 6[t] | 53 ± 6* |

a = 10.1 mg acetyl CoA formed/g wet weight
b = 5.27 mg acetyl CoA formed/ g wet weight
c = 9.2 mg citrate hydrolyzed/g wet weight
d = 9.17 mg citrate hydrolyzed/ g wet weight
e = 103020 dpm/g wet tissue f = 113322 dpm/g wet tissue
g = 86640 dpm/g wet tissue h = 64819 dpm/g wet tissue
i = 289450 dpm/g wet tissue j = 23099 dpm/g wet tiisue
k = 22443 dpm/g wet tissue l = 259099 dpm/g wet tissue
m = 43000 dpm/g wet tissue n = 54892 dpm/ g wet tissue
o = 87620 dpm/ g wet tissue p = 73219 dpm/g wet tissue
q = 11 ug Pi released /g wet tissue r = 111 ug Pi released/g wet tissue
s = 3112 dpm/g wet tissue t = 43128 dpm/g/ wet tissue
* = $P \geq 0.001$ Student's "t" test

TABLE 3
The Effects of Compound 4 on Sprague Dawley Male Rat Liver and Small Intestinal Mucosa Enzyme Activities After 14 Days at 8 mg/kg/day orally

| Enzyme Assay N = 6 | Percent Control [X ± S.D.] Liver | | Small Intestinal Mucosa | |
|---|---|---|---|---|
| | Control | Treated | Control | Treated |
| Acetyl CoA Synthetase | 100 ± 5 | 4 ± 2* | 100 ± 6 | 88 ± 6 |
| ATP Dep't Citrate Lyase | 100 ± 4 | 7 ± 4* | 100 ± 5 | 45 ± 7* |
| HMG CoA Reductase | 100 ± 6 | 50 ± 7* | 100 ± 7 | 117 ± 8 |
| Acyl Chol. Acyl Transferase | 100 ± 5 | 36 ± 2* | 100 ± 4 | 51 ± 8* |
| Cholesterol-7-Hydroxylase | 100 ± 3 | 69 ± 7* | 100 ± 4 | 65 ± 5* |
| Cholesterol Ester Hydrolase | 100 ± 4 | 8 ± 7* | 100 ± 5 | 237 ± 7* |
| Acetyl CoA Carboxylase | 100 ± 4 | 4 ± 7 | 100 ± 6 | 99 ± 5 |
| sn-Glycerol-3-phosphate Acyl Transferase | 100 ± 5 | 39 ± 5 | 100 ± 4 | 16 ± 4* |
| Phosphatidylate Phosphohydrolase | 100 ± 6 | 106 ± 6 | 100 ± 6 | 85 ± 6 |
| Lipoprotein Lipase | 100 ± 5 | 127 ± 4 | 100 ± 6 | 53 ± 6* |

See Table 2 for standard values for control assays lipids were within normal limits. Compound 4 afforded a 60% increase in biliary cholesterol and a 77% increase in biliary
phospholipid levels after 14 days. The bile salt concentrations were also altered after drug treatment, e.g. taurocholic, glycocholic, taurodeoxycholic, glycochenodeoxycholic and lithocholic acids were reduced by compound 1 while tauroursodeoxycholic, and glycourodeoxycholic acids were elevated and taurochenodeoxycholic acid was not changed from the control value. Compounds 4 reduced the levels of taurocholic, taurodeoxycholic, and glycourodeoxycholic acids.

Taurochendeoxycholic, glycocholic and tauroursodeoxycholic acids were elevated after treatment. Both compounds retarded cholesterol absorption from isolated intestinal loops after 180 min [Fig. 1 and 3]. Only compound 4 blocked cholic acid absorption from the isolated loops after 180 min [Fig. 2 and 4].

TABLE 4
The Effects of the Metal Complexes Of Amine-Carboxyboranes on Biliary Lipids and Bile Acids of Sprague Dawley Male Rats After 14 Days Administration Orally

| N = 6 | Bile Lipids mg/dL ± S.D. | | |
|---|---|---|---|
| | | Compound 1 | Compound 4 |
| Lipid | Control | 2.5 mg/kg/day | 8 mg/kg/day |
| Cholesterol | 113 ± 5 | 104 ± 4 | 181 ± 6* |
| Triglyceride | 140 ± 6 | 333 ± 12 | 123 ± 7* * |
| Neutral Lipids | 294 ± 8 | 297 ± 10 | 306 ± 11 |
| Phospholipids | 65 ± 4 | 66 ± 5 | 115 ± 7 |
| Protein | 97 ± 5 | 88 ± 8 | 96 ± |
| Flowrate ml/hr | 0.62 | 1.18 | 0.31 |

| | Bile Acids µg/ml ± S.D. | | |
|---|---|---|---|
| N = 6 | Control | Compound 1 | Compound 4 |
| Tauroursodeoxycholic Acid | 2.50 ±.08 | 6.54 ±.13 | 3.09 ±.10 |
| Glycoursodeoxycholic Acid | 561±.34 | 9.12 ±.54 | 3.35 ±.38 |
| Taurocholic Acid | 1.53 ±.06 | 0.01 ±.01 | 0.00 |
| Glycocholic Acid | 0.62 ±.04 | 0.02 ±.01 | 0.86 ±.03 |
| Taurodeoxycholic Acid | 0.50 ±.03 | 0.01±.01 | 0.26 ±.02 |
| Lithocholic Acid | 2.52 ±.15 | 1.01 ±.08 | 2.49 ±.11 |
| Taurochenodeoxycholic Acid | 0.24 ±.01 | 0.22 ±.02 | 0.52 ±.03 |
| Glycochenodeoxycholic Acid | 0.09 ±.01 | 0.01 ±.01 | 0.11 ±.02 |
| Total Bile Acids | 13.28 ± .58 | 16.94 ±.47 | 10.68 ±.32 |

### DISCUSSION

The copper, chromium, iron and zinc metal complexes of amine-carboxyboranes were generally more effective than sodium and cobalt complexes in reducing both serum cholesterol and triglyceride levels in mice after 16 days at 8 mg/kg/day I.P. The copper complex 1 also demonstrated good activity at 2.5 mg/kg/day, orally in rats after 14 days administration. These complexes were not the sterotype HMG CoA reductase enzyme inhibitor. Rather a number of regulatory enzymes for de novo synthesis of fatty acids, cholesterol, cholesterol esters and triglyceride were reduced by the complexes after in vivo administration in both the liver and small intestinal mucosa. The reduction of these enzyme activites by the agents was of a magnitude to account for the observed reduction of serum lipids afforded by the agents. One of the advantages of these complexes was the inhibition of acyl CoA cholesterol acyl transferase activity thus reducing the formation of cholesterol esters. Coupled with this effect of the agents was the accelerated activity of neutral cholesterol ester hydrolase afforded by the complexes which would lead to accelerated break down of cholesterol esters releasing free cholesterol to combine to HDL to return to the liver for excretion through the bile. If these enzymes are affected by the same manner in the aorta foam cells of the endothelium the formation of aorta plaques should be reduced as well as the incidence of atherosclerosis. A second mode of action of the metal complexes of amine-carboxyborane derivatives is the modulation of biliary excretion and enterohepatic circulation of lipids. Compound 1 increased bile flow and excretion of triglycerides whereas compound 4 accelerated excretion of cholesterol and phospholipids similiar to clofibrate effects. The bile acid profile was altered after drug treatment. However, there was no indication that large amounts of lithocholic acid was present which in rats is highly cholestatic [34]. Thus, there is no reason to think that these hypolipidemic agents may cause the formation of gall stones. Changes in bile acids can cause choleresis for example in decreasing order dehydrocholic > chenodeoxycholic > cholic acid > taurocholic acid > deoxycholic > glycocholic acid [35]. Compound 1 resulted in increases in glycourodeoxycholic acid and tauroursodeoxycholic acid which may have influenced the increase in bile flow observed for this agent. Compound 4 demonstrated no such large increases in any of the bile acids and no increase in bile flow. Gall stone formation has been linked with high levels of hepatic HMG CoA reductase activity and low levels of cholesterol-7-a hydroxylase activity. Neither compound created elevated HMG CoA reductase activity but both caused reduced cholesterol-7-a hydroxylase activity. This latter enzyme is the regulatory enzyme for the conversion of cholesterol to bile acids and should affect the bile acid profile if reduced in activity.

One mode of action of the metal complexes of amine carboxyborane derivatives was to block the resorption of cholesterol from the intestine thus returning it to the liver via the portal vein. Blocking this process is a viable mode of action of hypolipidemic agents, colestipol and cholestyramine, and would be additive with the other mode of action of the derivatives of inhibiting regulatory enzyme activities of de novo synthesis of lipids in the liver and small intestinal mucosa.

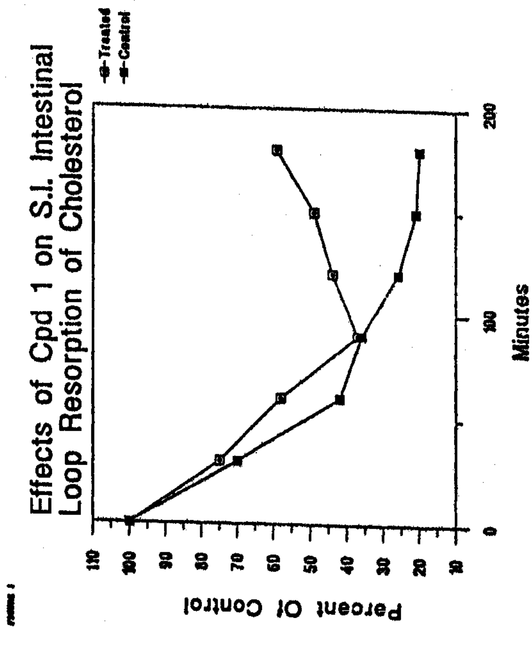

Effects of Cpd 1 on S.I. Intestinal Loop Resorption of Cholesterol

Percent Of Control

Minutes

-Treated

-Control

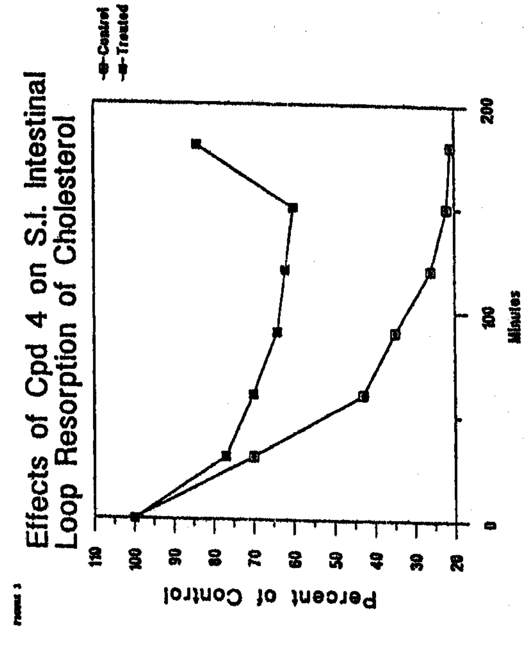

Effects of Cpd 4 on S.I. Intestinal Loop Resorption of Cholesterol

Percent of Control

Minutes

-Control

-Treated

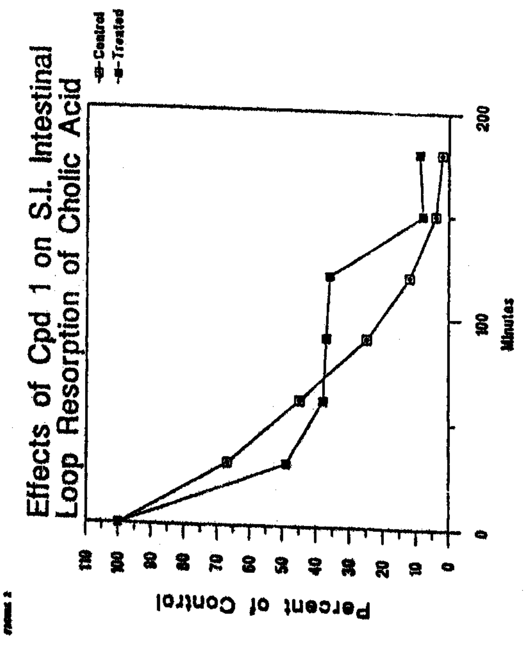

Effects of Cpd 1 on S.I. Intestinal Loop Resorption of Cholic Acid

Percent of Control

Minutes

-Control

-Treated

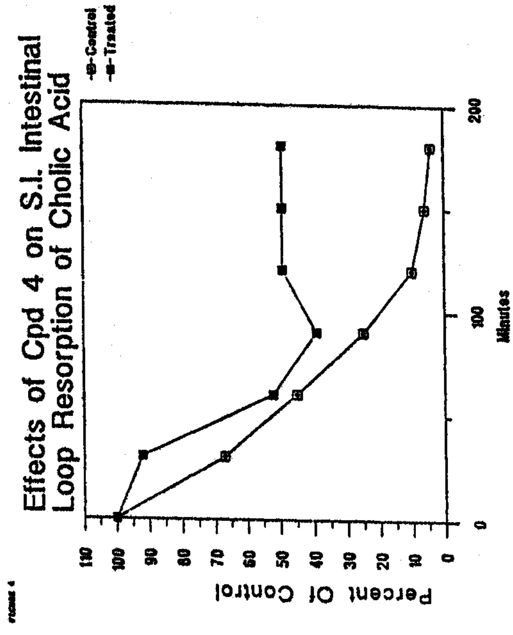

Effects of Cpd 4 on S.I. Intestinal Loop Resorption of Cholic Acid

Percent Of Control

Minutes

-Control

-Treated

### REFERENCES

1. Hall, I.H., Das, MK, Harchelroad, Jr., F, Wisian-Neilson, P, McPhail, AT, and Spielvogel,(1981) *J. Pharm. Sci.* **70**, 339-341.
2. Sood, A, Sood, CK, Spielvogel, BF, Hall, IH, and Wong, OT, (1991) *Arch Pharm.* **324**, 423-432.
3. Hall, IH, Spielvogel, BF, Griffin, TS, Docks, EL, and Brotherton, (1989) *Res. Commun. Chem. Path. Pharm.* **65**, 297-317.
4. Das, M.K. Maiti, PK, Roy, S, Mittakanti, M, Morse KW, Hall, IH, (1992) *Arch Pharm.* **325**, 267-272.
5. Hall, IH, Williams, Jr., WL, Gilbert, CJ, McPhail, AT, and Spielvogel, BF, (1984) *J. Pharm. Sci.* **73**, 973-977.
6. Hall, IH, Spielvogel, BF, McPhail, AT, (1984) *J. Pharm. Sci.* **73**, 222-225.
7. Norwood, VM, Morse, KW, (1986) *Inorg. Chem.* **25**, 3690-
8. Norwood, VM, Morse, KW, (1987) *Inorg. Chem.* **26**, 284-288.
9. Hall, IH, Gilbert, CJ, McPhail,(1984) *J. Pharm. Sci.* **74**, 765-768.
10. Hargrave, KD, McPhail, AT, Spielvogel, BF, Wisian-Neilson, P, (1977) *J. Chem. Soc., Dalton,* 2150-2153.
11. Hawthorne, MF, and Pitochelli, AR, (1959) *J. Am. Chem. Soc.* **81**, 5519.
12. Ness, A.T., Pastewka, J.V., and Peacock, A.C. (1964) *Clin. Chim. Acta.* **10**, 229-237.
13. Hall, I.H., Wong, O.T., Murthy, A.R.K., Day, P.A., and Calvin, J. (1987b) *Pharmacol. Res. Comm.* **19**, 839-858.
14. Bligh, E.G. and Dyer, W.J. (1959) *J. Biochem. Physiol.* **37**, 911-917.
15. Folch, J., Lees, M., and Stanley, G.H.C. (1957) *J. Biol. Chem.* **226**, 497-509.
16. Bragdon, J.H. (1951) *J. Biol. Chem.* 190, 513-517.17. Stewart, CP, Hendry, EG, (1935) *Biochem. J.* **29**, 1683-1689.
18. Lowry, O.H., Rosebrough, N.J., Farr, A.L., and Randall, R.J. (1951) *J. Biol. Chem.* **193**, 265-275.
19. Nakayama, F. and Nakagaki, M. (1980) *J. Chrom.* **183**, 287-293.
20. Hall, IH, Reynolds, DJ, Wong, OT, and Simlot, S, (1993) *Pharm. Res.* **27**, 129-139.
21. Doluisio, J.T., Tan, G.H., Billups, N.F., and Diamond, L. (1969) *J. Pharm. Sci.* **58**, 1200-1202.
22. Hall, I.H., Reynolds, D.J., Wong, O.T., Oswald, C.B., and Murthy, A.R.K. (1987) *Pharm. Res.* **4**, 472-479.
23. Hoffman, M., Weiss, L., and Wieland, O.H. (1978) *Anal. Biochem.* **84**, 441-448.
24. Goodridge, A.G. (1973) J. Biol. Chem. **248**, 4318-4327.
25. Haven, G.T., Krzemian, J.R., and Nguyen, T.T. (1973) *Res. Commun. Chem. Pathol. Pharmacol.* **6**, 253-261.
26. Wada, F., Hirata, K., and Sakameto, Y. (1989) *J. Biochem. Tokyo* **65**, 171-175.
27. Balsasubramaniam, S., Mitropoulos, K.A., and Venkatesan, S. (1978) *European J. Biochem.* **90**, 377-383.
28. Hall, I.H., Wong, O.T., Wyrick, S.D. (1988) *Pharm. Res.* **5**, 413-420.
29. Shefer, S., Hauser, S., Mosbach, E.H. (1978) *J. Lipid Res.* **9**, 328-337.
30. Greenspan, M.D. and Lowenstein, J.M. (1968) *J. Biol. Chem.* **243**, 6273-6280.
31. Lamb, R.G., Wyrick, S.D., Piantadosi, C. (1977) *Atherosclerosis* **27**, 147-154.
32. Mavis, R.D., Jacob, N., Finkelstein, J.N., and Hall, B.P. (1978) *J. Lipid Res.* **19**, 467-477.
33. Chait, A., Iverius, P.H., and Brunzell, J.D. (1982) J. Clin. *Invest.* 69, 490-493.
34. Turley, S.D. and Dietschy, J.M. (1979) *J. Lipid Res.* **20**, 923-934.
35. Bennion, L. and Grundy, S. (1978) *New Eng. J. Med.* **299**, 1161-1167.
36. Klaossen, C.D. and Watkins III, J.B. (1984) *Pharm. Res.* **36**, 1-67.
37. Kutz, K, Schulte, A, Just, C, Linstaedt, Reiter, B, (1979) *Arch Pharmacol.* **308**, 171-177.

**Received: September 9, 1993 - Accepted: October 10. 1993 - Accepted in camera-ready format: June 3, 1994**

